# Metabolic vulnerabilities and therapeutic opportunities in diffuse large B-cell lymphoma

**DOI:** 10.1038/s41389-026-00629-x

**Published:** 2026-05-15

**Authors:** Marie Anne-Catherine Neumann, Christian Frezza

**Affiliations:** 1 Department I of Internal Medicine, Center for Integrated Oncology Aachen Bonn Cologne Duesseldorf (CIO ABCD), University of Cologne, Faculty of Medicine and University Hospital of Cologne, University of Cologne, Cologne, Germany; 2 Faculty of Medicine and University Hospital Cologne, Institute for Metabolomics in Ageing, Cluster of Excellence Cellular Stress Responses in Aging-associated Diseases (CECAD), University of Cologne, Koln, Germany; 3 Faculty of Mathematics and Natural Sciences—Institute of Genetics, Cluster of Excellence Cellular Stress Responses in Aging-associated Diseases (CECAD), University of Cologne, Koln, Germany; 4 Center for Molecular Medicine (CMMC), University of Cologne, Cologne, Germany

**Keywords:** Cancer metabolism, Cell signalling

## Abstract

Diffuse large B cell lymphoma (DLBCL), the most common type of non-Hodgkin lymphoma (NHL), exhibits considerable biological heterogeneity. While its classification has traditionally relied on genetic and transcriptomic features, emerging evidence points to distinct metabolic subtypes that may represent novel therapeutic vulnerabilities. Intriguingly, current chemoimmunotherapy regimens exert profound but non-specific effects on tumour metabolism, inadvertently exploiting metabolic dependencies yet without precision. Novel inhibitors targeting glucose, amino acid, lipid, and mitochondrial metabolism demonstrate selective cytotoxicity in metabolically defined lymphoma subsets. This review investigates how standard therapies exploit DLBCL metabolism and examines heterogeneity across subtypes, and evaluates targeted metabolic therapies. We discuss emerging combination strategies with current therapeutic regimes and immunotherapy. Particular focus is given to the metabolic interactions between tumour cells and immune effectors, including CAR T cells and bispecific antibodies. We highlight the importance of translational research to validate metabolic subtypes through metabolomic profiling, identify predictive biomarkers, and develop rational combinations. Moving beyond empiric therapy towards strategic metabolic targeting offers an opportunity to enhance outcomes for patients with this aggressive and diverse lymphoma.

## DLBCL stratification through the lens of metabolism

Throughout their lifespan, B cells transition dynamically between states of quiescence and periods of intense metabolic activity. Germinal Centre (GC) B cells represent one of the most proliferative cell populations in the body, with a doubling time of approximately 6–8 h (Information Box) [1]. This rapid proliferation occurs within the uniquely challenging microenvironment of the GC, characterised by limited vascularisation and hypoxia, necessitating specific metabolic adaptations to sustain proliferation under such stress. Malignant transformation of GC B cells can give rise to diffuse large B cell lymphoma (DLBCL), the most common aggressive non-Hodgkin lymphoma (NHL) [2] and the focus of this review.

DLBCL exhibits profound heterogeneity across multiple biological dimensions. Historically, it has been classified based on transcriptomic profiling into germinal centre B cell-like (GCB) and activated B cell-like (ABC) subtypes, collectively referred to as the cell-of-origin (COO) classification [2]. Complementary genomic analyses have further delineated partially overlapping genetic subtypes characterised by distinct mutational landscapes and oncogenic drivers [3, 4]. While these classification systems have provided important insights into disease biology and prognosis, emerging evidence suggests that heterogeneity extends beyond genetic and transcriptomic features to encompass distinct metabolic programmes. Importantly, metabolic heterogeneity may represent an independent axis of biological diversity that is not fully captured by conventional classification schemes, potentially revealing previously unrecognised therapeutic vulnerabilities.

Expanding on transcriptional classification, Monti et al. [5] proposed a gene expression-based consensus cluster classification (CCC) that identifies subsets of DLBCL distinguished not only by signalling and differentiation programmes but also by predicted metabolic pathways. The ‘Oxidative Phosphorylation (OxPhos)’ cluster is enriched for genes associated with mitochondrial energy metabolism, oxidative phosphorylation, and fatty acid oxidation. In contrast, the ‘B cell receptor (BCR)/Proliferation’ cluster demonstrates transcriptional signatures consistent with glycolytic metabolism and BCR signalling. The ‘Host Response (HR)’ represents a distinct transcriptional subgroup characterised by upregulation of genes associated with immune activation, inflammatory signalling, and stromal interactions, reflecting the interplay between malignant B cells and their microenvironment. Of note, the CCC classification shows no correlation with COO subtypes [6], indicating that metabolic heterogeneity represents a distinct dimension of DLBCL biology that cannot be predicted from COO features alone. These findings collectively establish that DLBCL encompasses biologically discrete subsets with divergent metabolic dependencies that may serve as rational therapeutic targets.

It is essential to emphasise an important caveat with this approach: the CCC and similar classifications are based on transcriptomic data rather than direct metabolomic measurements. As such, the inferred metabolic programmes reflect predicted activity derived from gene expression patterns rather than actual metabolite fluxes, concentrations, or pathway activities. While transcriptional signatures provide valuable insights into potential metabolic phenotypes, they serve as proxies for metabolic activity and require validation through direct metabolomic profiling and functional assays. The correlation between metabolic gene expression and actual metabolic flux can be influenced by post-transcriptional regulation, modulation of enzyme activity, and substrate availability [7]. Future integrative studies combining transcriptomics, metabolomics, and functional metabolic flux analyses will be essential to validate these predictions, define the true extent of metabolic heterogeneity in DLBCL, and establish which metabolic features are clinically actionable. Nevertheless, the existing transcriptional data provide a compelling framework for hypothesis generation and suggest that metabolism-informed patient stratification may enhance therapeutic precision in this heterogeneous disease.

Box 1 B cell maturation in a box B-cell maturation is a highly ordered, multistep process that begins in the bone marrow, where hematopoietic stem cells differentiate into pro-B and pre-B cells. During these early stages, B cells undergo V(D)J recombination mediated by the RAG enzymes to assemble functional immunoglobulin heavy- and light-chain genes, generating a highly diverse B cell receptor (BCR) repertoire. Cells that successfully express a functional BCR and pass central tolerance checkpoints, eliminating or editing strongly self-reactive clones, exit the bone marrow. They then complete maturation in peripheral lymphoid tissues such as the spleen and lymph nodes, becoming (naïve) mature B cells.

Upon encountering cognate antigen, naïve B cells become activated and migrate into germinal centres (GC) within secondary lymphoid organs. Within the germinal centre, B cells undergo rapid clonal expansion in the dark zone, accompanied by somatic hypermutation of immunoglobulin variable regions, a process driven by activation-induced cytidine deaminase that introduces point mutations to increase antibody affinity. In the light zone, B cells undergo stringent selection based on their ability to bind antigen and receive survival signals from follicular helper T cells. Concurrently, class-switch recombination enables B cells to produce different antibody isotypes while retaining antigen specificity. Successfully selected cells differentiate into long-lived plasma cells or memory B cells.

The germinal centre reaction is inherently mutagenic, as it relies on DNA breaks and error-prone repair mechanisms. As a result, GC and post-GC B cells are particularly susceptible to oncogenic events. Diffuse large B-cell lymphoma (DLBCL) is believed to arise most frequently from B cells at these stages when normal regulatory mechanisms fail. Aberrant somatic hypermutation, chromosomal translocations involving oncogenes, and mutations in pathways governing BCR signalling, apoptosis, and cell cycle control can confer a growth and survival advantage. Accumulation of these genetic alterations ultimately drives malignant transformation and the development of DLBCL.

## Metabolic elements of standard care treatment for DLBCL

Current standard-of-care (SOC) therapies in DLBCL have significantly improved overall survival and cure rates for many patients. Nevertheless, roughly one-third of patients develop refractory or relapsed disease, underscoring a persistently poor prognosis and the pressing need for therapeutic innovation [8–10]. Insights from the CCC and other clusters highlight that DLBCL comprises biologically distinct subsets with divergent dependencies. This section examines how SOC treatments implicitly target tumour metabolism, impose metabolic stress, and reshape cellular energy pathways, thereby adding complexity to lymphoma therapy.

### First-line therapy

Historically, the therapeutic backbone for DLBCL has been polychemotherapy with cyclophosphamide, doxorubicin (hydroxydaunorubicin), vincristine (oncovin), and prednisone, collectively known as the CHOP regimen [11]. While initially designed to target proliferating cells through conventional cytotoxic mechanisms, each component of CHOP exerts profound metabolic stress on lymphoma cells. Cyclophosphamide, an alkylating agent, damages DNA and induces cell death, thereby forcing tumour cells to mobilise repair and survival pathways with high energetic costs [12]. Doxorubicin intercalates into DNA and generates reactive oxygen species, directly disrupting mitochondrial function and redox homoeostasis [13]. Vincristine interferes with microtubule assembly, impairing mitotic progression and altering cellular trafficking, processes that are tightly linked to energy metabolism [14]. The glucocorticoid prednisone induces apoptosis through transcriptional regulation of survival genes, but also reshapes glucose and lipid metabolism, both in malignant cells and in the systemic host environment [15, 16].

A breakthrough for DLBCL therapy occurred in 1998 with the introduction of rituximab, a monoclonal antibody targeting the B cell surface antigen CD20. When combined with CHOP chemotherapy (R-CHOP), rituximab markedly improved overall survival and progression-free survival compared to CHOP alone, firmly establishing R-CHOP as the standard first-line treatment for DLBCL [17]. Beyond its immunologic mechanisms, complement-dependent cytotoxicity, antibody-dependent cellular cytotoxicity, and antibody-dependent cellular phagocytosis, as well as direct signalling effects, rituximab has also been shown to modulate cellular metabolism. It can inhibit key survival pathways downstream of the B cell receptor (BCR), including PI3K/AKT and NF-κB, thereby suppressing glycolysis and reducing mitochondrial activity [18–20]. Despite the advances brought by R-CHOP and, more recently, the CD79b-targeting antibody-drug conjugate polatuzumab vedotin-containing regimens, a substantial fraction of patients still experiences refractory or relapsed disease [21]. Overcoming therapeutic resistance, or even anticipating which patients are predisposed to it, requires a deeper, more granular understanding of tumour biology. Among the multiple regulatory layers that drive disease heterogeneity and therapeutic response, cellular metabolism has emerged as a key determinant [22, 23]. Integrating metabolic features with treatment outcomes can be used to generate important new treatment strategies. DLBCL dependent on BCR signalling exhibit molecular signatures indicative of enhanced glycolytic flux and a strong dependency on BCR-mediated signalling pathways [24, 25]. In this context, the immunomodulatory action of rituximab, which can dampen PI3K/AKT and NF-κB signalling cascades, may confer additional therapeutic benefit by attenuating glycolytic activity and reducing metabolic support for proliferation. Furthermore, cyclophosphamide induces DNA crosslinking and replication stress, thereby increasing the energetic and biosynthetic demands associated with DNA damage repair. In glycolysis-enriched DLBCL, enhanced glucose uptake and glycolytic flux support rapid ATP generation as well as the provision of intermediates for nucleotide and redox metabolism. In principle, this metabolic configuration could influence the cellular response to alkylating agents, either by facilitating DNA repair through anabolic support or, conversely, by rendering highly proliferative cells more vulnerable to genotoxic stress. Prednisone, through glucocorticoid receptor signaling, induces profound metabolic rewiring, including glucose and lipid metabolism as well as transcriptional changes. Although these systemic and intracellular effects may interact with tumour metabolic states, direct evidence that glycolysis-enriched DLBCL derive preferential benefit from CHOP components remains lacking. Notably, the study by Monti et al. demonstrated comparable 5-year overall survival across the BCR, OxPhos, and HR metabolic clusters in CHOP-treated patients, arguing against a clearly differential therapeutic response based solely on metabolic classification [5]. In contrast, OxPhos-enriched lymphomas rely more strongly on mitochondrial oxidative phosphorylation, mitochondrial translation, and redox homoeostasis for survival [25]. For these metabolically distinct tumors, therapeutic regimens that exacerbate mitochondrial stress or disrupt redox homoeostasis may be particularly effective. Agents such as doxorubicin, which induce reactive oxygen species (ROS) and compromise mitochondrial function, or polatuzumab vedotin, which interferes with intracellular protein trafficking and biosynthesis [26], may further exploit intrinsic vulnerabilities of OxPhos-dependent malignancies. Collectively, these observations indicate that R-CHOP represents a collateral ‘metabolic cocktail’ that may achieve differential efficacy based on tumour metabolic rewiring.

### Relapsed or refractory DLBCL

The advent of cellular and bispecific immunotherapies has driven a transformative shift in the management of relapsed or refractory DLBCL. Chimeric antigen receptor (CAR) T cell therapy redirects autologous T cells to recognise CD19 on malignant B cells. Beyond their potent cytolytic effects, CAR T cells impose a distinct metabolic pressure on tumour cells by inducing profound immune-mediated stress [27, 28]. Engagement of CAR T cells triggers perforin/granzyme-dependent killing, interferon-γ secretion, and inflammatory cytokine release, collectively remodelling the tumour microenvironment and altering metabolic substrate availability [29]. Moreover, sustained T cell activation itself depends on robust glycolytic and mitochondrial function, and emerging data suggest that the metabolic fitness of both the CAR T cells and the tumour cells can influence therapeutic efficacy and resistance [30–32]. Tumour cells with high OxPhos capacity or enhanced antioxidant defences may better withstand cytokine-induced oxidative stress, whereas glycolysis-dominant tumours might be more susceptible to immune-mediated metabolic competition.

#### Metabolic features of CAR T cells

The metabolic characteristics of CAR T cell products themselves represent a critical determinant of therapeutic efficacy, persistence, and resistance. T cells undergo profound metabolic reprogramming during activation and differentiation: naïve and central memory subsets rely primarily on OxPhos and fatty acid oxidation (FAO) to support longevity and quiescence, whereas effector and effector memory subsets are predominantly glycolytic, favouring rapid proliferation and cytotoxic function but with limited persistence [30, 33]. Consequently, CAR T cell products enriched in stem cell memory subsets exhibit superior in vivo expansion and long-term tumour control compared to products dominated by glycolysis-driven effector cells [34].

The manufacturing process itself, particularly cytokine milieu and nutrient availability during ex vivo expansion, also shapes the metabolic imprint of the CAR T product [35]. IL-2–driven cultures favour glycolytic effector differentiation, while IL-7 and IL-15-based protocols promote FAO and memory formation [36, 37]. Glucose-rich media encourage mTORC1 activation and short-lived effector states, whereas glucose restriction or pharmacologic modulation of glycolysis can bias CAR T differentiation toward long-lived, metabolically flexible cells with enhanced persistence [32, 38–40].

The choice of the co-stimulatory domain within the CAR construct imposes distinct metabolic and transcriptional programmes. CD28-based CARs (e.g., axicabtagene ciloleucel) promote a glycolytic, highly activated phenotype with rapid effector differentiation, increased glucose transporter 1 (GLUT1) expression, and higher mTORC1 activity. This supports potent initial cytotoxicity but is often associated with early exhaustion and reduced persistence. In contrast, 4-1BB-based CARs (e.g., tisagenlecleucel and lisocabtagene maraleucel) preferentially engage PGC-1α and AMPK signalling, driving mitochondrial biogenesis and spare respiratory capacity. This metabolic bias supports oxidative metabolism, memory formation, and enhanced persistence. These metabolic distinctions are paralleled by differences in exhaustion profiles: glycolytic CD28-CAR T cells show higher expression of inhibitory receptors (PD-1, TIM-3, LAG-3), whereas OxPhos-dominant 4-1BB CAR T cells maintain a more stem-like, polyfunctional phenotype [41–44].

These findings indicate that the metabolic phenotype of the infused CAR T product, shaped by both intrinsic T cell subset composition and CAR signalling architecture, can profoundly affect therapeutic kinetics. Products dominated by glycolytic effector cells may achieve rapid tumour debulking but are prone to exhaustion, whereas OxPhos-reliant, memory-like CAR T cells demonstrate superior durability and recall capacity. Significantly, the metabolic profile of the target lymphoma itself may further influence which CAR T cell phenotype performs optimally. For example, lymphomas with a glycolytic, highly proliferative signature may be more efficiently cleared by CAR T products with strong early effector activity, despite a shorter persistence window. Conversely, tumours, which rely more on OxPhos and on mitochondrial metabolism, often reside in metabolically restrictive or immunosuppressive niches and may preferentially respond to CAR T cells with enhanced mitochondrial fitness, oxidative flexibility, and resistance to nutrient competition or hypoxia. This concept supports the rationale for matching CAR T metabolic programmes to tumour metabolic phenotypes, integrating tumour biology into product design and patient selection. Future strategies to metabolically engineer CAR T cells by modulating PGC-1α, AMPK, or mitochondrial dynamics may therefore enhance both persistence and resilience in the hostile metabolic landscape of the lymphoma microenvironment, enabling precision pairing of CAR design with tumour subtypes.

#### Bispecific T cell engagers

Bispecific T cell engagers (BiTEs) are a type of artificial antibody that simultaneously binds to an effector cell protein (e.g., CD3 in T cells) and a tumour cell surface protein. This off-the-shelf approach enables to direct cytotoxic T cells toward CD20-expressing lymphoma cells [45]. BiTE-induced immune synapse formation provokes a rapid and sustained T cell activation that leads to direct cytolysis and cytokine release, generating a microenvironmental context similar to CAR T therapy but with different kinetics and reversibility [46, 47]. The continuous engagement between T cells and tumour cells creates intense local metabolic competition for glucose, amino acids, and oxygen [48, 49]. This metabolic tug-of-war can profoundly shape therapeutic outcomes. Lymphoma cells with flexible metabolic programmes, capable of adapting to shifts in nutrient availability or oxidative stress, may be better able to withstand immune-mediated attack. In contrast, tumours with rigid or highly specialised metabolic wiring may be less able to compensate under conditions of immune-driven nutrient deprivation, rendering them more susceptible to T cell-mediated cytotoxicity. These insights underscore the potential of integrating metabolic profiling into the design of immune-based therapies. By identifying tumours with specific metabolic vulnerabilities, it may be possible to rationally design combination strategies, such as pairing BiTEs with metabolic inhibitors or immune modulators, to enhance antitumor efficacy and overcome resistance mechanisms.

Taken together, the established SOC for DLBCL relies not only on direct cytotoxic and immune-mediated effects but also on metabolic stress imposed on malignant B cells. This recognition underscores that metabolism is already an implicit therapeutic target in DLBCL, suggesting that a more deliberate exploitation of metabolic vulnerabilities could complement or enhance existing treatment strategies. The emergence of immune-based approaches further reinforces the idea that metabolic plasticity is not merely a hallmark of lymphoma biology but also a key determinant of treatment response. Integrating tumour- and T cell-specific metabolic features provides a conceptual bridge between conventional and immune-directed modalities, offering a framework for rational combination strategies. Metabolic modulators could be paired with CAR T or BiTE therapies to unmask hidden vulnerabilities. Ultimately, a deeper integration of metabolic phenotyping with immunotherapy may lay the foundation for a metabolism-informed precision treatment paradigm in DLBCL.

## Improvement of current strategies: new experimental metabolic targets and treatments in DLBCL

**Fig. 1:**
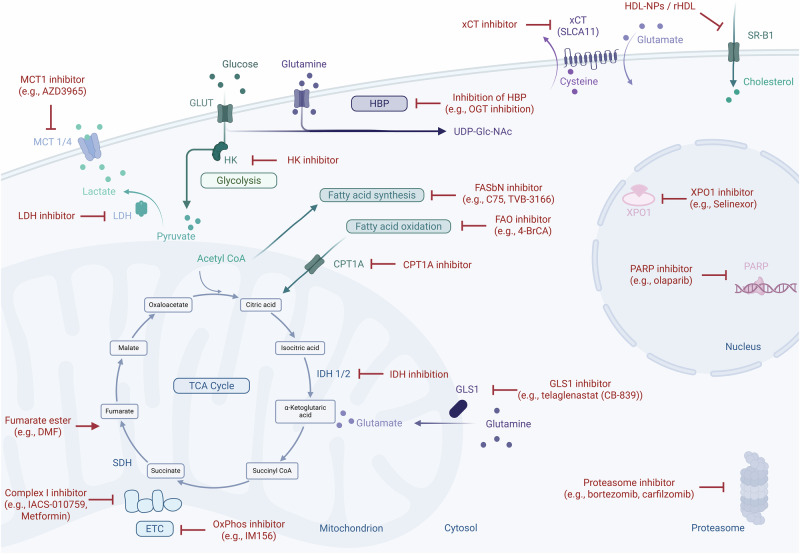
Schematic overview: new strategies of metabolic targets in DLBCL. Abbreviation: Acetyl CoA Acetyl coenzyme A, CPT1A Carnitine palmitoyltransferase 1A, DMF Dimethyl fumarate, FAO Fatty acid oxidation, FASbN Fatty acid synthase β-subunit N-terminal domain, GLS Glutaminase, GLUT Glucose transporter, HBP Hexosamine biosynthetic pathway, HDL-NPs High-density lipoprotein nanoparticles, HK – Hexokinase, IDH Isocitrate dehydrogenase, LDH Lactate dehydrogenase, MCT1 Monocarboxylate transporter 1, OGT O-GlcNAc transferase, OxPhos Oxidative phosphorylation, PARP Poly(ADP-ribose) polymerase, rHDL Reconstituted high-density lipoprotein, SDH Succinate dehydrogenase, SR-B1 Scavenger receptor class B type 1, UPD-Glc-NAC Uridine diphosphate N-acetylglucosamine, xCT Cystine/glutamate antiporter (SLC7A11), XPO – Exportin; Figure created with www.BioRender.com.

Building on the concept that metabolism represents a therapeutic vulnerability in lymphoma, the next challenge is to translate metabolic insights into novel treatment paradigms and precision medicine strategies to improve outcomes. The distinct metabolic signatures of DLBCL not only provide a framework for biological and clinical stratification but also unveil opportunities to exploit subtype-specific metabolic dependencies as actionable therapeutic targets. Targeting these metabolic pathways could therefore (i) leverage intrinsic vulnerabilities unique to specific DLBCL subtypes, (ii) intensify cellular stress induced by chemotherapy or immunotherapy, and (iii) remodel a tolerogenic tumour microenvironment into one that is permissive to effective immune-mediated clearance (Fig. 1, Table 1).

**Table 1:** Metabolic targets in DLBCL clinical studies.

Target (Agent)	Mechanism	Study	
MCT1 inhibitor (AZD3965)	Inhibition of lactate influx or efflux	Phase I (NCT01791595)	monotherapy in rrDLBCL and Burkitt lymphoma: 11 patients Safety and tolerability: proven safe in humans Efficacy: 10% CR (15 months), 10% SD, 80% no response
GLS1 inhibitor (Telaglenastat, CB-839)	Inhibition of glutaminolysis and TCA anaplerosis	Phase I (NCT02071888)	monotherapy in advanced and or refractory haematological malignancies completed, not reported yet
XPO1 inhibitor (Selinexor, KPT-330, ATG-010)	Inhibition of nuclear export and disruption of proteostasis	Phase IIb (NCT02227251) SEDAL trail	monotherapy in rrDLBCL still recruiting
		Phase II (NCT05422066)	combination with R-CHOP in high-risk GCB-subtype DLBCL still recruiting
		Phase Ib/II (NCT05577364)	combination with R-CHOP and Selinexor maintenance in EBV-positive DLBCL still recruiting
		Phase II (NCT06517511), Smart trail	combination with R-CHOP in untreated TP53-mutated DLBCL still recruiting
		Phase II/III (NCT04442022)	combination with GDP (SR-GDP) in rrDLBCL still recruiting
PARP inhibitor (Olaparib)	Inhibition of Poly(ADP-ribose) polymerase and Induction of DNA single and double strand breaks	Phase I (NCT03259503)	Combination with high-dose chemotherapy (GemBuMel) for rrLymphomas Safety and tolerability: proven safe in humans Efficacy: 100% ORR with 90% CR (30 months FU); EFS 72%; OS 82%
Biguanide (Metformin)	Inhibition of complex I and activator of AMPK	Phase II (NCT02531308)	Combination with R-CHOP of previously untreated DLBCL terminated due to poor patient accrual
		Phase II (NCT02815397)	Combination with DA-EPOCH-R in double hit DLBCL terminated due to poor patient accrual
Proteasome inhibitors (Bortezomib)	Inhibition of protein degradation and induction of proteotoxic stress	Phase II (NCT00066508)	monotherapy of bortezomib in rrDLBCL Safety and tolerability: no unexpected safety signals unique to DLBCL Efficacy: Rare durable CR
		Phase III (NCT01324596), REMoDLB trail	Combination with R-CHOP in previously untreated DLBCL Safety and tolerability: no significant increase of toxicity compared to R-CHOP Efficacy: no significant difference compared to R-CHOP; subgroup benefit in ABC-DLBCL (5-year-OS: 80% vs 67%)
		Phase II (NCT01805557)	Combination with R-DHAP in transplant-eligible rrDLBCL Safety and tolerability: no significant increase of toxicity compared to R-DHAP Efficacy: no significant difference compared to R-DHAP
		Phase I/ II (NCT03129828), ImbruVeRCHOP	Combination with Ibrutinib and R-CHOP in previously untreated DLBCL completed, not fully reported yet
		Phase II (NCT00931918)	Combination with R-CHOP in non-GCB DLBCL Safety and tolerability: no significant increase of toxicity compared to R-CHOP Efficacy: no significant difference compared to R-CHOP
		Phase II (NCT00504751), Viper	Combination with ifosfamide, cisplatin, etoposide, rituximab and dexamethasone in rrDLBCL, 15 patients Safety and tolerability: feasible regime with relevant toxicity Efficacy: not superior to existing options
		Phase II (NCT01848132)	Combination with R-CHOP in young high-risk DLBCL Safety and tolerability: no significant increase of toxicity compared to R-CHOP Efficacy: no significant difference compared to R-CHOP
		Phase II (NCT01040871)	Combination with vincristine, rituximab, cyclophosphamide, doxorubicin and prednisolone (VR-CAP) in previously untreated DLBCL Safety and tolerability: no significant increase of toxicity compared to R-CHOP Efficacy: no significant difference compared to R-CHOP

*ABC*-*DLBCL* Activated B-cell–like Diffuse Large B-Cell Lymphoma, *AMPK* AMP-activated Protein Kinase, *BTK* Bruton’s Tyrosine Kinase, *CR* Complete Respons, DA-EPOCH-R Dose-Adjusted Etoposide, Prednisone, Vincristine, Cyclophosphamide, Doxorubicin + Rituximab, DLBCL Diffuse Large B-Cell Lymphoma, *EBV* Epstein–Barr Virus, *EFS* Event-Free Survival, *FU* Follow-Up, *GCB* Germinal Center B-cell–lik, *GDP* Gemcitabine, Dexamethasone, Cisplatin, GemBuMel Gemcitabine, Busulfan, Melphalan, GLS1 Glutaminase 1, *MCT*1 Monocarboxylate Transporter 1, *NCT* National Clinical Trial identifier (ClinicalTrials.gov), ORR Overall Response Rate, OS Overall Survival, PARP Poly(ADP-ribose) Polymerase, R-CHOP Rituximab, Cyclophosphamide, Doxorubicin, Vincristine (Oncovin), Prednisone, R-DHAP Rituximab, Dexamethasone, High-dose Cytarabine, Cisplatin, rrDLBCL Relapsed or Refractory Diffuse Large B-Cell Lymphoma, *SD* Stable Disease, *TCA* Tricarboxylic Acid (cycle), *TP5*3 Tumour Protein p53 gene, *XPO*1 Exportin-1.

### Targeting glucose and amino acid metabolism

Monocarboxylate transport (*MCT*) and lactate metabolism represent critical metabolic dependencies in subsets of DLBCL. Many DLBCL tumours rely heavily on aerobic glycolysis, generating large quantities of lactate that must be exported to maintain intracellular pH and sustain high glycolytic flux. This process is mediated primarily by the monocarboxylate transporter MCT1 (SLC16A1) [50, 51]. Genetic or pharmacologic inhibition of MCT1, most notably using the selective inhibitor AZD3965, results in intracellular lactate accumulation, NAD⁺ depletion, impaired glycolytic ATP production, and growth arrest in MCT1-high/MCT4-low DLBCL cell lines. Preclinical models demonstrate that MCT1 blockade triggers metabolic crisis and cell death and enhances sensitivity to therapies that further restrict oxidative capacity, including mitochondrial complex I inhibitors and agents that elevate reactive oxygen species. DLBCL subtypes lacking compensatory MCT4 expression show the most significant vulnerability, establishing a biomarker-defined therapeutic niche [52–54]. Early clinical evaluation of AZD3965 supports the feasibility of targeting MCT1 in lymphoma. A first-in-human phase I study (NCT01791595) in advanced solid tumours and lymphomas has reported acceptable tolerability at biologically active doses, on-target lactate modulation, and preliminary signals of anti-lymphoma activity, including disease stabilisation in heavily pretreated DLBCL [55]. Ongoing dose-expansion cohorts are refining patient selection based on MCT1/MCT4 expression and metabolic biomarker profiling.

Parallel efforts to target lactate metabolism at the level of lactate dehydrogenase (*LDH*) have elucidated complementary vulnerabilities. LDHA catalyses the conversion of pyruvate to lactate, regenerating NAD⁺ to sustain glycolytic flux. In DLBCL models, LDHA inhibition reduces NAD⁺ availability, impairs ATP production, elevates mitochondrial ROS, and induces apoptotic and non-apoptotic cell death. LDH blockade synergises with MCT1 inhibition by simultaneously preventing lactate production and export, pushing glycolytic tumour cells into severe redox and energetic collapse [56, 57]. Although LDH inhibitors remain preclinical, they lead to potent and selective anti-tumour responses in in vitro models, advancing toward early-phase development [57].

Hexokinases (*HKs*) catalyse the first committed step of glycolysis, phosphorylating glucose to glucose-6-phosphate and thereby trapping glucose within the cell. In DLBCL, increased expression and mitochondrial association of hexokinase isoforms, particularly hexokinase II (HK2), support high rates of glucose uptake and glycolytic flux characteristic of aggressive disease [58]. Mitochondria-bound HK2 couples glycolysis directly to ATP generated by OxPhos and confers an additional survival advantage by interacting with the voltage-dependent anion channel, thereby limiting mitochondrial outer membrane permeabilisation and apoptosis [59]. Functional studies in lymphoma models demonstrate that disruption of hexokinase activity or its mitochondrial localisation reduces glycolytic throughput, depletes ATP, sensitises cells to oxidative stress, and lowers the apoptotic threshold. These effects are especially pronounced in DLBCL subtypes with MYC-driven metabolic reprogramming [58, 60], establishing hexokinase as both a metabolic gatekeeper and a pro-survival factor in DLBCL.

Beyond its canonical function in glycolysis, glyceraldehyde-3-phosphate dehydrogenase (*GAPDH*) has emerged as a critical metabolic and regulatory node in aggressive lymphomas. GAPDH catalyses the conversion of glyceraldehyde-3-phosphate to 1,3-bisphosphoglycerate with concomitant reduction of NAD⁺ to NADH, thereby directly coupling glycolytic flux to the cytosolic NAD⁺/NADH ratio and redox homoeostasis [61]. Under conditions of mitochondrial dysfunction or hypoxia, where electron transport chain–dependent NAD⁺ regeneration is impaired, multiple quantitative flux analyses have shown that GAPDH assumes rate-limiting control over glycolysis such that partial inhibition of GAPDH is sufficient to reduce overall glycolytic throughput and ATP production [61, 62]. It was demonstrated that *GAPDH–low* DLBCL tumours exhibit a distinct metabolic programme characterised by reduced glycolytic ATP production, increased reliance on OxPhos, heightened mTORC1 signalling, and enhanced glutaminolysis, whereas GAPDH–high tumours retain robust glycolytic activity and better responsiveness to glycolysis-targeted therapies. Significantly, low GAPDH expression predicted poor response to standard R-CHOP therapy and indicated selective vulnerabilities to metabolic interventions such as phenformin, L-asparaginase, and mTOR inhibition, which exploit the OxPhos dependence of these cells. Consequently, GAPDH expression and activity represent actionable biomarkers and potential targets [63].

Malignant cells characteristically display increased glucose uptake, accompanied by heightened glycolytic activity and glucose transport. While most glucose is funnelled through glycolysis, roughly 5% is shunted toward the hexosamine biosynthetic pathway (*HBP*). By integrating glucose and glutamine availability, the HBP regulates O-GlcNAcylation, a post-translational modification that stabilises transcription factors such as NF-κB and maintains pro-survival signalling [64]. Inhibition of O-GlcNAc transferase (OGT) or upstream HBP enzymes reduces O-GlcNAcylation, destabilises transcription factors, and induces cell-cycle arrest in DLBCL models, highlighting the potential of targeting nutrient-sensitive post-translational modifications. These findings suggest that HBP inhibitors may be particularly effective in DLBCL subsets with high glucose and glutamine flux, warranting further clinical investigation [65].

Glutamine metabolism is a central axis in many DLBCL tumours. Glutamine fuels TCA cycle anaplerosis, supports nucleotide and lipid biosynthesis, and maintains redox homoeostasis via glutathione production [66, 67]. Pharmacologic inhibition of glutaminase (*GLS1*) disrupts the conversion of glutamine to glutamate, reducing the supply of α-ketoglutarate (αKG) for the TCA cycle, increasing mitochondrial ROS, and inducing both apoptotic and non-apoptotic cell death. GLS1 blockade also sensitises DLBCL cells to BCL-2 antagonists and other agents by lowering the threshold for mitochondrial apoptosis [67–69]. Complementary metabolic vulnerabilities exist in cysteine metabolism: Cysteine availability is critical for glutathione synthesis and redox homoeostasis in DLBCL. Although a generalised inability of DLBCL cells to synthesise cysteine via the trans-sulfuration pathway cannot be assumed, cystine uptake through the system xCT transporter (SLC7A11) represents an important mechanism regulating intracellular redox balance in multiple cancer entities. Inhibition of xCT disrupts cystine import, depletes glutathione, and can induce ferroptosis, an iron-dependent form of lipid peroxidation-driven cell death [70]. Notably, ferroptosis resistance mechanisms, including glutathione peroxidase 4 (GPX4), have been implicated in DLBCL biology [71, 72], suggesting that redox regulation constitutes a relevant vulnerability axis. While transcriptomic datasets indicate that SLC7A11 expression in DLBCL is variable and not uniformly elevated, context-dependent reliance on cystine uptake cannot be excluded. Thus, targeting xCT-mediated cystine import may represent a potential therapeutic strategy in selected metabolic or redox-defined DLBCL subsets and warrants further functional validation ^72^. Early-phase clinical studies of the oral GLS1 inhibitor telaglenastat (CB-839) in mainly solid cancers have demonstrated safety and biological activity. An open-label Phase 1 study (NCT02071888) is testing the effects of CB-839 in subjects with haematological tumours, and results have not yet been reported for lymphoma. Ongoing combination trials, including CAR T cells, bispecific antibodies, and checkpoint inhibitors, exploit metabolic-immune synergies [73–75]. Although single-agent activity has been modest, these studies provide a framework for metabolism-immune co-targeting strategies in glutamine-addicted DLBCL subtypes.

### TCA cycle

The TCA cycle is central to B cell energy metabolism, biosynthesis, and redox homoeostasis, and its dysregulation in DLBCL creates multiple potential therapeutic vulnerabilities.

Citrate generated by citrate synthase sustains TCA cycle flux, supporting energy production and redox balance. It also serves as a critical substrate for de novo lipid biosynthesis through its export to the cytosol and cleavage by ATP-citrate lyase (*ACLY*) into acetyl-CoA and oxaloacetate, thereby linking mitochondrial metabolism to the de novo lipid biosynthesis and signalling lipids essential for rapid proliferation and membrane expansion in aggressive subtypes [76–78]. Transcriptomic and metabolomic analyses reveal elevated citrate export and ACLY activity, particularly in GCB-DLBCL and BCR-driven models, where mitochondrial pyruvate import fuels citrate accumulation that supports fatty acid synthesis for ER/Golgi biogenesis and oncogenic signalling via lipid second messengers, with glutamine contributing prominently to citrate via reductive carboxylation in OxPhos-dependent subsets [6, 79–81].

Isocitrate dehydrogenase 1/2 (*IDH1/2*) catalyse the oxidative decarboxylation of isocitrate to α-KG, generating NADPH essential for redox defence, lipid synthesis, and biosynthetic reactions in proliferating cells. Although canonical neomorphic IDH1/2 mutations producing 2-hydroxyglutarate are rare in DLBCL (<5% of cases), unlike in AML or gliomas, wild-type IDH1/2-linked oxidative metabolic flux is upregulated in OxPhos-dependent DLBCL subsets, particularly GCB- and MYC-driven tumours. In these contexts, glutamine anaplerosis fuels isocitrate production sustaining NADPH generation to support glutathione recycling and thioredoxin systems that counteract oxidative stress from BCR signalling or MYC-induced replication [6, 82–84]. Beside direct IDH inhibition, modulation of αKG is being explored in DLBCL. αKG serves as a central anaplerotic metabolite and as an essential cofactor for dioxygenases that control epigenetic regulation and redox homoeostasis. Glutaminase inhibitors limit glutamine-to-αKG conversion, starving OxPhos-high cells of anaplerotic fuel and provoking reductive stress. GPX inhibitors deplete glutathione-dependent defences and disrupt ferroptosis thresholds; thereby bypassing classical BLC2/MYC-mediated apoptosis resistance in DLBCL [85–88].

The TCA intermediate fumarate is produced by succinate dehydrogenase (*SDH*), an enzyme that uniquely links the TCA cycle to the electron transport chain (ETC). SDH dysfunction can lead to fumarate accumulation and elevated ROS production, thereby disrupting redox homoeostasis and sensitising lymphoma cells to mitochondrial OxPhos inhibitors. This highlights a potential vulnerability in OxPhos-dependent lymphoma subtypes, where pharmacologic modulation of SDH activity could selectively impose metabolic and oxidative stress [89, 90]. Notably, fumarate esters, such as dimethyl fumarate (DMF) and its bioactive metabolite monomethyl fumarate (MMF), can mimic aspects of fumarate accumulation and exert pleiotropic effects on tumour metabolism and immune signalling. Beyond their established clinical use in multiple sclerosis and psoriasis, DMF and MMF have been shown to modulate cellular redox balance, glutathione metabolism, and NF-κB–dependent inflammatory pathways. [91–94]. In the lymphoma context, these agents could act as metabolic stress inducers, overwhelming antioxidant defences in metabolically inflexible tumours, or reprogramme the tumour microenvironment toward a more immunostimulatory state [72]. Recent preclinical data in acute myeloid leukaemia (AML) demonstrate that DMF markedly increases intracellular ROS and reduces mitochondrial respiration. It synergises with the BCL-2 inhibitor venetoclax to induce mitochondrial depolarisation and apoptosis, including in venetoclax-resistant AML models [95]. Thus, therapeutic fumarate ester-induced metabolic perturbation represents an emerging strategy that may complement existing chemo- or immunotherapeutic approaches in metabolically defined (refractory) lymphoma subsets.

Malate and oxaloacetate (OAA) are critical TCA cycle intermediates that drive the malate-aspartate shuttle, transferring cytosolic NADH into mitochondria for OxPhos while enabling aspartate export for nucleotide biosynthesis, which is essential for the rapid proliferation of OxPhos-dependent DLBCL subsets [80, 96]. Plasma metabolomics from newly diagnosed DLBCL patients revealed decreased aspartate levels correlating with poor prognosis and diagnostic signatures, indicating aspartate limitation as a marker of active disease that distinguishes DLBCL from complete remission [81]. Malate accumulates as an unfavourable prognostic biomarker, positively correlating with tumour metabolic volume and total lesion glycolysis, while isotope-tracing confirms glutamine-derived malate/ OAA fuelling aspartate synthesis via GOT2/MDH2 in BCR- and ECM-driven DLBCL models [6, 80, 81].

Collectively, the TCA intermediates and their associated enzymes represent a network of metabolic dependencies in B cells and lymphoma. Targeting them, either alone or in combination with mitochondrial stressors, redox modulators, or immune therapies, offers a rational approach to exploit metabolic vulnerabilities in DLBCL.

### OxPhos and ETC

DLBCL subsets with OxPhos signatures, including MYC-driven or double-hit tumours, are heavily reliant on mitochondrial respiration. Inhibition of the mitochondrial ETC, particularly *complex I*, collapses ATP production, disrupts the NADH/NAD⁺ balance, and provokes lethal oxidative stress in cells unable to switch rapidly to glycolysis [6, 25]. The complex I inhibitor IACS-010759 has shown on-target inhibition of ETC activity, preliminary antitumor responses, and a tolerable safety profile in first-in-human phase I studies (NCT03291938, NCT02882321) in patients with advanced solid tumours and haematologic malignancies, thereby providing clinical proof-of-concept that pharmacologic OxPhos blockade is feasible in humans [97, 98]. A separate first-in-human study of IM156, a next-generation, potent biguanide OxPhos inhibitor, similarly demonstrated acceptable safety, evidence of ETC target engagement, and disease control in a subset of patients with advanced solid tumours (NCT05497778, NCT03272256), reinforcing the translational viability of this therapeutic class [99]. Preclinical studies indicate that combining OxPhos inhibitors with BCL-2 antagonists produces synergistic cytotoxicity by simultaneously amplifying mitochondrial stress and lowering the threshold for intrinsic apoptosis. Furthermore, simultaneous inhibition of mitochondrial complex I and the MCT1 showed synergistic tumour cell killing by concurrently disrupting both mitochondrial respiration and lactate export, leading to cell death in models where either agent alone was only cytostatic [100–102]. Strategically integrating OxPhos-directed agents with other metabolic agents or immunotherapies represents a promising translational avenue to exploit mitochondrial dependencies and enhance antitumor immunity in metabolically defined DLBCL subsets.

Notably, recent studies have demonstrated that lymphoma and other cancer cells can adapt to targeted therapies or chemotherapy by shifting from glycolytic metabolism toward increased mitochondrial respiration, thereby enhancing ATP production, redox buffering capacity, and survival under treatment-induced stress [103, 104]. Such adaptive OxPhos upregulation may contribute to resistance against agents that primarily target proliferative or glycolysis-dependent states. These findings underscore the dual role of mitochondrial respiration in DLBCL, as both a metabolic vulnerability in intrinsically OxPhos-dependent subsets and an adaptive survival pathway under therapeutic pressure. Consequently, early integration of OxPhos inhibitors into combination regimens may not only exploit baseline mitochondrial dependencies but also prevent or overcome therapy-induced metabolic rewiring.

### Lipid metabolism

Fatty acid metabolism adds an additional layer of vulnerability. Several DLBCL models exhibit a pronounced dependence on fatty acid oxidation (FAO) as a key source of ATP and NADPH, particularly under conditions of glucose or glutamine limitation. This metabolic flexibility enables tumour cells to sustain energy production and redox balance within a nutrient-scarce microenvironment [79, 105]. Pharmacologic or genetic inhibition of FAO induces energetic stress, enhances oxidative damage, and sensitises tumours to chemotherapeutic agents [79, 106]. FAO can be inhibited by using the small-molecule FAO-inhibitor 4-bromocrotonic acid (4-BrCA), or through targeting the carnitine palmitoyl transferase 1A (*CPT1A*), the rate-limiting mitochondrial fatty acid transporter for long-chain fatty acid import into mitochondrial ß-oxidation. CPT1A is overexpressed in lymphoid malignancies and correlates with poor outcome or therapy resistance [107–110]. However, despite promising preclinical biology, clinical development of irreversible CPT1 inhibitors has been hampered by off-target toxicity in human studies [111]. As a result, there are no approved CPT1A inhibitors for lymphoma and only limited clinical testing in non-oncology indications; caution is warranted for systemic CPT1 inhibition. This is a major reason the field is exploring more selective, reversible inhibitors or alternative ways to target FAO.

Concurrently, fatty acid synthase (*FASbN*), the rate-limiting enzyme in fatty-acid synthesis, is found frequently overexpressed in DLBCL, correlating with poor clinical outcomes and driving oncogenic signalling networks, including c-Met-mediated pathways and mechanisms that inhibit ferroptosis [105, 112, 113]. Pharmacologic blockade of FASN using inhibitors such as C75 or TVB-3166 induces potent, dose-dependent cytotoxicity in DLBCL models, characterised by impaired lipid synthesis, disruption of membrane-associated signalling platforms, mitochondrial dysfunction, and enhanced oxidative and apoptotic stress. These lipid-metabolic perturbations synergise with other mitochondrial and apoptotic stressors, underscoring a therapeutically actionable dependence on lipogenesis in DLBCL [110, 114]. In addition, lipid metabolism is increasingly recognised as a central regulator of ferroptosis susceptibility in DLBCL linking fatty acid synthesis, lipid remodelling, and antioxidant defence systems, such as GPX4-mediated detoxification of lipid peroxides, to redox homoeostasis and treatment response [115, 116]. Thus, targeting lipogenesis may not only impair tumour growth but also shift the balance toward lipid peroxidation-induced ferroptotic cell death. Beyond intrinsic lipid synthesis, exogenous lipid acquisition also contributes to lymphoma progression. Fatty acid translocase, CD36, facilitates lipid uptake and supports tumour growth. Elevated FASN and CD36 expression were shown to predict chemoresistance in rituximab-treated DLBCL [117, 118].

Innovative therapeutic strategies are increasingly leveraging lipid-metabolic dependencies in B-cell malignancies. High-density lipoprotein–mimetic nanoparticles (HDL-NPs/rHDL) can bind the HDL receptor SR-B1 on malignant B cells. Promoting cellular cholesterol efflux or blocking cholesterol uptake can induce selective apoptosis in B-cell lymphoma models [119]. In lymphoma models that resist cholesterol depletion via compensatory de novo synthesis, combining HDL-NPs with inhibitors of proximal BCR-signalling reduces intracellular cholesterol more effectively, disrupts lipid-raft integrity, and attenuates BCR-dependent prosurvival signalling. Mechanistically, disruption or cholesterol depletion of plasma-membrane lipid rafts is well established to impair clustering and function of BCR signalosomes and downstream AKT/SYK/BTK signalling, providing a plausible basis for the observed synergy with kinase inhibitors [120–123].

Collectively, these findings highlight lipid metabolism as a multifaceted vulnerability in DLBCL. Therapeutic strategies that co-target these pathways, particularly in combination with chemotherapy or immunotherapies, could selectively compromise lipid-dependent lymphoma subsets and enhance treatment efficacy.

### Pleiotropic metabolic modulators

#### Nuclear export and DNA repair dependencies

Agents targeting nuclear export are currently being explored in clinical settings. Selinexor, a selective inhibitor of nuclear export (*XPO1*), exemplifies this approach. By blocking the export of tumour suppressor proteins, oncogenic transcription factors, and metabolic regulators from the nucleus, selinexor induces proteostatic stress, disrupts cellular energy balance, and triggers apoptosis through metabolic and oxidative stress pathways [124, 125]. In the multicenter, open-label Phase 2b SADAL trial, selinexor demonstrates single-agent efficacy in relapsed or refractory DLBCL, establishing proof-of-concept that non-cytotoxic, orally bioavailable agents with pleiotropic metabolic consequences can achieve meaningful clinical benefit [126]. Furthermore, four ongoing phase II and III studies (NCT05422066, NCT05577364, NCT04442022, NCT06517511) are recruiting patients to evaluate selinexor in combination with polychemotherapy regimens. All four trials remain active and recruiting as of late 2025, focusing on improving outcomes in both frontline high-risk and RR DLBCL through XPO1 inhibition with standard chemotherapies.

Poly(ADP-ribose) polymerase (*PARP*) inhibitors block PARP-mediated base excision repair and related DNA damage response pathways, thereby exploiting genomic instability and high replication stress. Beyond enforcing the accumulation of DNA single- and double-strand breaks, PARP inhibition depletes and perturbs cellular NAD⁺ pools and can impair mitochondrial function, thereby functionally coupling genotoxic stress to metabolic vulnerability in tumour cells with limited metabolic plasticity [127, 128]. Importantly, emerging data suggest that PARP inhibitor sensitivity in DLBCL may not be uniform but confined to biologically defined subgroups. Parvin et al. [129] identified a subset of DLBCL characterised by expression of LIM domain only 2 (LMO2), associated with defective homologous recombination repair and enhanced sensitivity to olaparib. These findings indicate that homologous recombination deficiency-like features may stratify patients who derive particular benefit from PARP inhibition. In addition, metabolic rewiring may further modulate PARP inhibitor sensitivity. Recent work demonstrated that DLBCL cells resistant to R-CHOP and transiently responsive to L-asparaginase undergo adaptive metabolic reprogramming toward enhanced serine/glycine synthesis [130]. This shift promotes nucleotide biosynthesis and redox buffering but unexpectedly creates a therapeutically actionable vulnerability to PARP inhibition. These findings underscore the intimate coupling between metabolic plasticity and DNA repair dependencies in DLBCL and further support the rationale for integrating PARP inhibitors into metabolically stratified treatment strategies. These properties provide a rationale for combining PARP inhibitors with therapies that further compromise bioenergetics, where convergent stress on DNA repair and mitochondrial function enhances cytotoxicity in metabolically inflexible lymphoma subtypes and may intensify immunogenic cell death signals through increased DNA damage–associated inflammatory signalling. In a first-in-human phase I trial (NCT03259503), the PARP inhibitor olaparib was combined with polychemotherapy and autologous stem-cell transplantation in relapsed or refractory lymphomas, including DLBCL. The olaparib-containing regimen was feasible, identified a recommended phase II dose, and showed high response rates with encouraging event-free and overall survival in this high-risk population [99].

#### Metabolic adjuvants

Beyond targeted export inhibition, metabolic adjuvants such as metformin, a well-characterised *AMPK* activator, have been investigated as combinatorial partners with chemotherapy and immunotherapy. Metformin and related biguanides reprogramme tumour bioenergetics by inhibiting mitochondrial complex I, thereby lowering ATP production, increasing AMP/ATP ratios, and activating catabolic pathways that oppose anabolic tumour growth. In lymphoma models, these effects can synergise with cytotoxic or immune-based therapies, sensitising metabolically rigid tumour cells to stress and potentially enhancing T-cell function through microenvironmental reconditioning [131, 132]. Two phase II studies (NCT02531308 and NCT02815397) investigated the role of metformin combined with polychemotherapy in the frontline and relapse setting for DLBCL. Both studies were terminated early due to poor patient accrual. Notably, dual inhibition of glucose transport with ritonavir, which blocks glucose uptake via GLUT4, inducing a compensatory shift toward OxPhos, and inhibition of OxPhos with metformin reduces proliferation and survival in myeloma and chronic lymphocytic leukaemia (CLL) (NCT02948283). The findings provide a mechanistic rationale for combining metformin or next-generation biguanides with agents that block glucose transport in lymphoma, particularly those with limited capacity to flexibly switch between glycolysis and OxPhos.

*PI3K* inhibitors target critical metabolic and survival pathways by blocking the PI3K/AKT/mTOR axis, thereby reducing glucose uptake via GLUT transporters, suppressing glycolytic flux, impairing lipid and nucleotide biosynthesis, and disrupting pro-survival signalling in malignant B cells. FDA-approved for CLL and follicular lymphoma, PI3K inhibition enhances the efficacy of chemotherapeutic agents or targeted therapies in DLBCL by exploiting metabolic vulnerabilities, lowering the apoptotic threshold through BIM upregulation, and preventing compensatory anabolic signalling, which frequently mediates resistance to single-agent treatments. These effects are particularly pronounced in ABC DLBCL subsets, which exhibit constitutive PI3K pathway activation and reliance on mTOR-driven protein synthesis for survival. By overcoming the metabolic inflexibility of malignant B cells, PI3K inhibitors restore sensitivity to conventional therapies [133–135].

*Proteasome* inhibitors such as bortezomib and carfilzomib induce proteotoxic stress by blocking protein degradation, triggering ER stress and the unfolded protein response. This proteotoxic stress is highly metabolically demanding, as it requires upregulated protein folding chaperones and lipid synthesis to expand the ER membrane; thereby creating potential synergies with agents that impair mitochondrial OxPhos or lipid metabolism. Proteasome inhibition can also enhance tumour immunogenicity by promoting immunogenic cell death and increasing antigen presentation when combined with immunotherapies. The clinical relevance of these mechanisms is underscored by the REMoDL-B phase III trial (NCT01324596), which demonstrated that the addition of bortezomib to R-CHOP (RB-CHOP) could improve PFS and OS specifically in ABC-DLBCL subtypes. These subtype-specific outcomes align with the heightened reliance of ABC-DLBCL on NF-κB-driven protein turnover and ER stress tolerance, positioning proteasome inhibitors as rational metabolic adjuvants in precision frontline regimens for metabolically vulnerable DLBCL subsets [136–138].

## Current challenges for metabolic targeting in DLBCL

While targeting metabolic vulnerabilities in DLBCL offers promising therapeutic opportunities, several practical challenges and toxicity considerations must be addressed. Many core metabolic enzymes are shared between tumour and healthy tissues, meaning that on-target toxicity can affect several organs and tissues. Careful dose-finding and schedule optimisation are therefore critical to maximise the therapeutic window. Another key challenge is tumour metabolic plasticity: DLBCL cells can adaptively rewire their metabolism, shifting between glycolysis, OxPhos or FAO under therapeutic pressure. This dynamic adaptability argues for combination or sequential treatment strategies that target multiple pathways or exploit metabolic dependencies at specific vulnerabilities [139, 140].

A further challenge is inter- and intra-tumour metabolic heterogeneity. Distinct cellular subpopulations within the same tumour may engage different metabolic programmes according to local microenvironmental constraints, including oxygen and nutrient availability. This functional diversification can permit partial escape from pathway-targeted interventions. Addressing this will likely require combinatorial approaches alongside emerging single-cell and spatially resolved profiling strategies to identify context-specific metabolic liabilities. The tumour microenvironment, including stromal cells, immune infiltrates, together with extracellular nutrient availability, can buffer metabolic stress, allowing lymphoma cells to survive single-agent metabolic interventions. Pharmacodynamic monitoring using pathway-specific biomarkers can help identify early responders and guide therapy adjustments, ultimately improving the precision and durability of metabolic-targeted approaches.

The metabolic heterogeneity of DLBCL defines a network of actionable vulnerabilities that can be therapeutically exploited. Rationally designed combination regimens integrating metabolic inhibitors with chemotherapy or immune-based approaches offer promising avenues to enhance efficacy and overcome resistance in DLBCL. The integration of metabolic biomarkers into clinical trial design provides a path to precision-targeted therapy in DLBCL. Patients can be stratified based on tumour-intrinsic metabolic profiles to predict sensitivity to targeted therapies [81, 141, 142]. Complementary metabolomic assays can provide functional readouts of metabolic flux and redox stress. At the same time, advanced imaging modalities, such as FDG-PET dynamics or emerging mitochondrial PET tracers, allow non-invasive assessment of metabolic activity in real time. Integrating microenvironmental biomarkers can help to identify patients who benefit from combinations of metabolic inhibitors with chemo- and immunotherapies [143].

## Conclusions and future perspective

This review provides evidence that metabolic phenotypes are not merely passive consequences of oncogenic transformation but active determinants of therapeutic response, resistance, and immune evasion. Indeed, across DLBCL subtypes, from glycolysis-dominant, BCR-dependent lymphomas to OxPhos-reliant, mitochondria-driven malignancies, metabolic rewiring creates both vulnerabilities and adaptive mechanisms that profoundly influence treatment outcomes. This metabolic heterogeneity of DLBCL, shaped by distinct genetic drivers, microenvironmental pressures, and therapeutic interventions, represents a critical yet underexploited dimension of tumour biology. While direct clinical validation is still lacking, the framework underscores the key concept that therapeutic efficacy in DLBCL is determined not only by genetic and immunologic features but also by the tumour’s metabolic rewiring. We propose that refining this perspective with integrated metabolomic, transcriptomic, and functional data is imperative to move toward metabolism-guided treatment stratification and resistance prediction.

Future strategies are likely to integrate multi-omic profiling and functional imaging to define patient-specific metabolic vulnerabilities and guide therapy selection. Advances in metabolic engineering of immune therapies may allow modulation of T cell bioenergetics to enhance persistence and efficacy within metabolically hostile tumour microenvironments. The development of adaptive, biomarker-driven clinical trials will be essential to test combinations of metabolic inhibitors with chemotherapy or immunotherapy, optimising dosing, scheduling, and sequencing to exploit tumour metabolic plasticity while minimising toxicity. Emerging targets expand the therapeutic landscape and offer opportunities to overcome resistance in refractory or high-risk disease. Ultimately, integrating metabolic insights with immunologic and genomic profiling promises a paradigm shift in DLBCL treatment, moving toward fully personalised, metabolism-informed therapeutic strategies that maximise efficacy, limit toxicity, and improve long-term patient outcomes.
